# Gangrène congénitale entité rare en néonatalogie: à propos d'une observation

**DOI:** 10.11604/pamj.2019.33.59.16710

**Published:** 2019-05-28

**Authors:** Anass Ayyad, Sahar Messaoudi, Rim Amrani

**Affiliations:** 1 Service de Néonatalogie, Centre Hospitalier Universitaire Mohamed VI, Faculté de Médecine et de Pharmacie d'Oujda, Université Mohammed Premier, Oujda, Maroc

**Keywords:** Gangrène congénitale, ischémie aiguë, sepsis, membre supérieur, Congenital gangrene, acute ischemia, sepsis, upper limb

## Abstract

La gangrène néonatale est une entité rare en néonatalogie dont le pronostic est généralement mauvais. L'ischémie aiguë des membr es est le plus souvent causée par des phénomènes thromboemboliques. Bien qu'il existe plusieurs facteurs de prédisposition, dans la majorité des cas, aucun facteur étiologique n'est retrouvé. L'étendue de la gangrène est également variable, allant d'un ou plusieurs doigts ou orteils à l'ensemble du membre supérieur ou inférieur. Nous rapportons un cas de gangrène congénitale de la main colligé dans notre formation.

## Abstract (en)

Neonatal gangrene is a rare condition during the neonatal period. Its prognosis is generally poor. Acute ischemia of the limbs is most often caused by thromboembolic events. Although there are several predisposing factors, in the majority of cases no causative factor is found. Also the extent of the gangrene is variable; it can affect one or several fingers or toes or even the whole upper or lower limb. We here report a case of congenital gangrene of the hand treated in our Department.

## Introduction

La gangrène congénitale des extrémités du nouveau-né est rarissime en néonatalogie son étiologie est obscure et dans de nombreux cas, aucun facteur étiologique n'est mis en évidence [1, 2]. Néanmoins, toute gangrène néonatale suggère une étiopathogenèse intra-utérine, plusieurs facteurs de risque peuvent être liés à la gangrène congénitale. On en cite: la prématurité, l'infection systémique, le diabète maternel, les présentations dystociques, l'hypothermie, cardiopathies congénitale. La prise en charge n'est pas codifiée et le traitement se base sur l'antibiothérapie, l'héparinothérapie et l'ambulation chirurgicale. Nous rapportant un cas de gangrène congénitale de la main colligé dans notre service.

## Patient et observation

**Figure 1:**
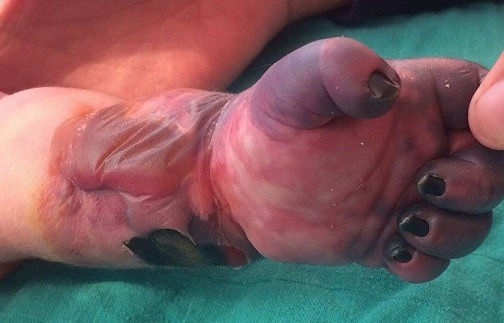
Image de la face palmaire de la main montrant la nécrose et son étendu

**Figure 2:**
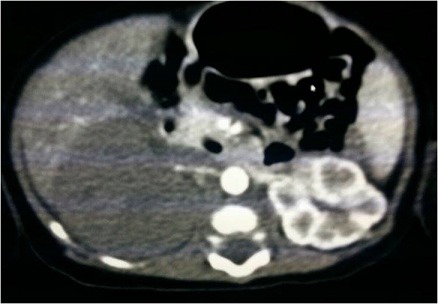
Coupe axiale angio-TDM objectivant le néphrome mésoblastique

Il s'agit d'un nouveau-né (n-né) de sexe masculin issu d'un mariage non consanguin et d'une mère âgée de 24 ans, primipare. Adressé à notre formation pour prise en charge d'une cyanose de la main gauche. La grossesse a été bien suivie et d'évolution apparemment normale. L'accouchement s'est déroulé par voie haute après stagnation de la dilatation pendant 5 heures chez une femme primipare, avec présentation de siège. L'Apgar a été de 7/10 et 9/10 à 1 min et 5 min respectivement. Dès la naissance, le nouveau-né a présenté une phlyctène qui s'est aggravée 48h plus tard par une cyanose totale de la main gauche, d'où la décision de son transfert dans notre formation pour complément de prise en charge. L'examen clinique à l'admission a trouvé un n-né stable sur les plans: hémodynamique, neurologique et respiratoire, pesant 4300g, avec une nécrose de la main et de la moitié de l'avant-bras gauche (Figure 1). Le bilan biologique a montré un syndrome infectieux, avec un taux de prothrombine (TP) à 60% et un temps de céphaline activée (TCA) normal. L'angio-TDM a mis en évidence une thrombose de l'artère humérale au niveau du tiers inférieur du bras gauche, et un néphrome mésoblastique au dépond du rein gauche avec envahissement de la veine rénale homolatérale (Figure 2). Le n-né a été mis sous triple antibiothérapie avec des soins locaux. Un traitement chirurgicale été programmé mais l'évolution a été marquée par le décès 24 heures après l'admission dans un contexte d'état de mal convulsif dû probablement à une migration d'un thrombus en intra-cérébral.

## Discussion

La gangrène des extrémités chez le nouveau-né est extrêmement rare, les cas rapporté par les auteurs se comptent sur les bouts des doigts [1-7]. L'ischémie aiguë des membres est le plus souvent causée par des phénomènes thromboemboliques [1, 6]. Plusieurs facteurs de risque tel que le diabète gestationnel, la prématurité, les cardiopathies congénitales, la prématurité, les traumatismes obstétricaux, la polyglobulie, septicémie etc& ont été cités dans la littérature comme facteurs de prédisposition à la gangrène congénitale des extrémités, cependant aucun facteur étiologique n'est retrouvé dans la majorité des cas [1, 3, 4]. La prise en charge de ces nouveaux nés comporte deux volets: le premier est un traitement médical à base d'antibiothérapie associée ou non à une héparinothérapie. Le second est chirurgical mais vu l'impact psychosocial majeur pour le patient et sa famille ainsi que le pouvoir de régénération tissulaire du nouveau-né [2, 8]. On tend vers la temporisation en ne procédant pas à l'amputation d'emblée dans l'attente d'une démarcation définitive de la partie gangrenée mais en cas de gangrène établie, l'amputation est le traitement de choix. Pourtant, en revoyant la littérature, on a retrouvé que presque tous les cas rapportés ont procédé à une amputation urgente [3-5]. Par contre, Timothy et col ont rapporté un cas de thrombectomie réalisé à H10 de vie chez un nouveau né ayant une nécrose de la main [6]. Ainsi Ufuk Aydin et col ont traité avec succès un cas de gangrène congénitale du membre supérieur grâce à la thérapie à pression négative [8].

## Conclusion

La gangrène congénitale reste une entité rarissime en néonatalogie; seulement une dizaine de cas ont été rapportés par la littérature. La physiopathologie de ces cas de gangrène congénitale reste mal établie, de même que l'étiologie. La prise en charge thérapeutique fait l'objet de controverses entre ceux qui préconisent l'amputation d'emblée et ceux qui penchent plus vers un traitement conservateur en un premier temps.

## Conflits d’intérêts

Les auteurs ne déclarent aucun conflit d'intérêts.
